# A Novel Linear Model Based on Code Approximation for GNSS/INS Ultra-Tight Integration System

**DOI:** 10.3390/s20113192

**Published:** 2020-06-04

**Authors:** Zhe Yan, Xiyuan Chen, Xinhua Tang

**Affiliations:** Key Laboratory of Micro-Inertial Instrument and Advanced Navigation Technology, Ministry of Education, School of Instrument Science and Engineering, Southeast University, Nanjing 210096, China; seuyanzhe@163.com (Z.Y.); xinhuatanggnss@163.com (X.T.)

**Keywords:** GNSS/INS, ultra-tight integration, nonlinear measurement, code deviation

## Abstract

The superiority of a global navigation satellite system (GNSS)/inertial navigation system (INS) ultra-tight integration navigation system has been widely verified. For those systems with centralized structure based on coherent-accumulation measurements (I/Q), the conversion from I/Q signals to navigation information is implemented by an observation equation. As a result, the model is highly complex and nonlinear, exerting essential influence on system performance. Based on the analysis of previous studies, a novel model and its linearization method are proposed, aiming at the integrity, stability and implicit nonlinear factors. Unlike the one-order precision in the common Jacobian matrix, two-order components are partly reserved in this model, which makes it possible for higher positioning accuracy and better convergence. For the positioning errors caused by ignoring code-loop deviation, a method to approximate code-phase is proposed without introducing new measurements. Consequently, the effect of code error can be significantly reduced, especially when the tracking loops are unstable. In the end, using real-sampled satellite signals, semi-physical experiments are carried out and the effectiveness and superiority of new methods are proved.

## 1. Introduction

The complementary characteristics between a global navigation satellite system (GNSS) and inertial navigation system (INS) have been widely used in GNSS/INS integration systems [1]. In loose integration mode, GNSS and INS work independently to output positioning information which is then fused directly via a navigation filter [2]. More primitive information is used in a tightly integrated system, where the errors of pseudo-range and pseudo-range rate are used as observations [1]. In some tightly integrated architectures, carrier-phase measurements are also introduced as observations [3]. Positioning accuracy and robustness can be improved in both integrated modes than any single system [4], but the corrections obtained from the integration filter are all for INS and useless in improving the performance of the GNSS receiver. This decoupling may make the tracking loop of the GNSS receiver the weakest link in the whole system, and too sensitive to work in weak signal and high-dynamic scenarios.

Ultra-tight integration based on vector tracking is an effective method proposed for the above problems [5], and its main idea differs from conventional scalar loops. By fusing the information from tracking channels and INS, tracking errors are estimated to correct carrier and code generators to form a new closed loop. State-of-the-art, ultra-tightly integrated systems can be classified into different schemes according to various data-fusion degrees and filter structures. Herein, ultra-tight integrations based on vector tracking are considered to use coherent-accumulation results as basic observations and are categorized as federated and centralized schemes. In federated structure, pre-filters are used for each channel to calculate Doppler frequency and code-phase error using in-phase (I) and quadra-phase (Q) coherent accumulation results. In addition, an integration filter is involved to establish the relationship between pre-filters’ outputs and the errors of positioning. In the end, corrections for INS and the feedbacks for tracking loops can be constructed. In theory, the pre-filters function as discriminators with the advantage in overcoming the limitation of linear range when using conventional discriminators. Generally, variations based on such federated frameworks are diverse in practical applications. For instance, scalar discriminators can still be used instead of pre-filters [6,7,8], but the integration filter shares the same way with tight integration on the basis of pseudo-ranges and pseudo-range rates [8,9,10,11]. Then along the line-of-sight vector, the feedbacks of tracking loops are generated. Another approach is that the results of pre-filters can be directly fed back to numerically controlled oscillators (NCOs), or a combined method of the above two is also useful [12,13,14,15]. A detailed implementation of federated structure is introduced by Ohlmeyer [16], in which the filter is designed to estimate INS errors but not receiver errors. Besides, INS plays no significant role in filtering, and the implementation is similar to a tracking loop aided by INS in theory. Therefore, a more ideal approach when designing a federated filter is to use the information estimated by INS, including pseudo-ranges and pseudo-range rates [8,10,17], or Dopplers and code-phases [18]. 

In centralized architecture, a mathematical model is established directly between positioning information and coherent-accumulation measurements (I/Q), so that a predicted I/Q can be obtained from INS and the differences between predicted and actually received I/Q can be utilized as observations [19]. Great importance should be attached to this method because the I/Q is closer to Gaussian distribution than other information in tracking channels. This character is consistent with the Gaussian hypothesis of the Kalman filter. In a centralized approach, pre-filters are unnecessary and only one integration filter is needed. However, a main problem that comes with centralized architecture is the complex and highly non-linear model, because an integration filter completes the data conversion in a single step. As a result, the precision of positioning relies on the accuracy of the measurement model and the processing of non-linearity to a great extent.

Currently, many researches on model linearization and non-linear filtering for the GNSS/INS system have been done [19,20,21,22,23,24,25]. Among them, Babu et al. takes the lead in giving the detailed model of centralized architecture and verifying it using simulated signals [19]. By theoretical analysis, the problem that the observation equation of the model does not satisfy is the stability condition, which may lead to filter divergence, which was improved by Chen et al. [26]. However, the linearization method for the observation model provided by [26] makes it that I signals can no longer be used as measurements. So, the north velocity and height were selected as observation variables to ensure the integrity. Jwo et al. studied the non-linear filtering algorithms for this model, and the performance of non-linear algorithms based on Extended Kalman Filter (EKF) and Unscented Kalman Filter (UKF) were compared [20,21]. Cubature Kalman Filter (CKF) is potentially useful, too, but with a higher computation cost [22,23,24,25]. According to the linearization methods provided by those papers, the observation equation in EKF seems to be a linear expression of position error $x_{e}$ and velocity error ${\dot{x}}_{e}$. Actually, the errors of frequency and initial phase, included in some seeming constants, are functions of $x_{e}$ or ${\dot{x}}_{e}$ inherently. Therefore, strong nonlinearity is still implicit in observation equation, and the Jacobian matrix obtained has not been completely simplified, which may lead to large errors or even filter divergence. The same shortcomings can be also seen in the research of Zhou et al. [27,28].

In addition, due to the neglecting of code error when constructing the I/Q model, the estimation of position is obtained from the initial carrier phase [19,29]. In practice, the main source of position error is the deviation of code loop. The assumption is that a precisely locked code loop will inevitably lead to a larger error in positioning, and only carrier parameters are fused with INS. So, the deviation is neither conducive to INS correction nor modifiable because of inaccurate feedback to the vector-tracking code loop. In the case of using real sampled I/Q signals instead of simulated ones, or when I/Q signals are not ideal, position error and filtering divergence caused by model bias are particularly evident. As a consequent, a conventional scalar code tracking loop is still relied on.

The main work and contributions of this research can be summarized as follows. Firstly, a novel linearization method for the measurement equation is proposed to recalculate the Jacobian matrix, in which second-order accuracy can be achieved in some components. The positioning accuracy of ultra-tight integration system can be effectively improved. Another benefit is that a standard Kalman filter can be used rather than complicated non-linear algorithms. This decrease in computation cost is quite meaningful in practical application. Secondly, a novel model based on code approximation is also presented to narrow position error. Without introducing new observations, the burden of filter is not increased. Thirdly, the validity of such new approaches is verified under the circumstance of loop oscillation. The results illustrate an apparent improvement on position precision, making it possible for the realization of vector tracking. In the end, the stability of filtering is analyzed and verified.

## 2. Integration Model Based on Scalar Code Loops

**Figure 1:**
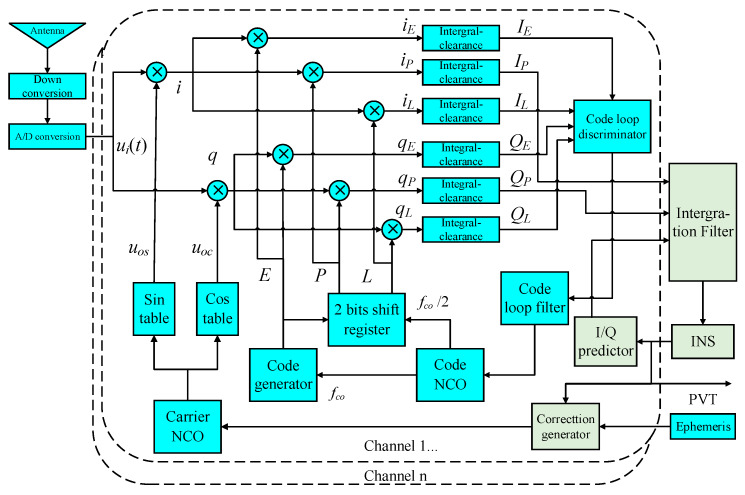
Centralized ultra-tight integration system based on scalar code loops.

The ultra-tight integration system in Figure 1 is firstly introduced by Babu [19] and was widely discussed immediately among many scholars. In Figure 1, *I_P_*/*Q_P_*, *I_E_*/*Q_E_*, and *I_L_*/*Q_L_* are the I/Q signals of prompt, early and late branches, respectively, and their code phase interval with each other is half a code chip. The basic principle is to utilize the information provided by INS to estimate I/Q signals of the receiver, and the differences between I/Q measurements from receiver channels and former estimated ones are regarded as observations. Meanwhile, using modified positioning information and ephemeris, the parameters for loop control can be calculated to constitute a vector-tracking carrier loop, but a scalar code loop is still used and not contributory in data fusion. The main problems are about the establishment of I/Q estimation and how to construct the observation equation properly.

Suppose $\omega_{e}$ and $\theta_{e}$ denotes Doppler frequency error and initial carrier phase error, respectively. Calculating the expectations of I/Q in prompt branch gives

(1) $$E\left[I_{P}\right]=\frac{A}{2\omega_{e}}[\sin\left(\omega_{e}\left(k+1\right)T+\theta_{e}\right)-\sin\left(\omega_{e}kT+\theta_{e}\right)];$$

(2) $$E\left[Q_{P}\right]=-\frac{A}{2\omega_{e}}[\cos\left(\omega_{e}\left(k+1\right)T+\theta_{e}\right)-\cos\left(\omega_{e}kT+\theta_{e}\right)],$$

where k means kth sample, and T is sampling interval. Furthermore, carrier frequency error and initial phase error can be correlated with position error $R_{e}$ and velocity error $v_{e}$ as follows

(3) $$\omega_{e}=\frac{\omega }{c}\left|v_{u}-{\hat{v}}_{u}\right|=\frac{\omega }{c}v_{e};$$

(4) $$\theta_{e}=-\frac{\omega }{c}\left[\left|R_{u}-{\hat{R}}_{u}\right|-\left|v_{u}-{\hat{v}}_{u}\right|t\right]=-\frac{\omega }{c}\left[R_{e}-v_{e}t\right],$$

where $R_{u}$ and ${\hat{R}}_{u}$ represent, respectively, the measured and estimated position vectors, while $v_{u}$ and ${\hat{v}}_{u}$ are velocity vectors. *c* is the speed of light. Let $R_{e}$ and $v_{e}$ be the Euclidean distances of position errors and velocity errors in three directions, given by

(5) $$R_{e}=\sqrt{x_{e}^{2}+y_{e}^{2}+z_{e}^{2}}=\sqrt{{\left(\hat{x}-x\right)}^{2}+{\left(\hat{y}-y\right)}^{2}+{\left(\hat{z}-z\right)}^{2}};$$

(6) $$v_{e}=\sqrt{{\dot{x}}_{e}^{2}+{\dot{y}}_{e}^{2}+{\dot{z}}_{e}^{2}}=\sqrt{{\left(\hat{\dot{x}}-\dot{x}\right)}^{2}+{\left(\hat{\dot{y}}-\dot{y}\right)}^{2}+{\left(\hat{\dot{z}}-\dot{z}\right)}^{2}}.$$

Then, according to Formulas (1)–(6), the estimation of I/Q can be obtained.

In the GNSS/INS integration system, the state vector of Kalman filter can be expressed as a 17-dimensional vector, given by

(7) $$x_{k}={\left[\delta x,\delta y,\delta z,\delta \dot{x},\delta \dot{y},\delta \dot{z},\nabla_{x},\nabla_{y},\nabla_{z},\varphi_{x},\varphi_{y},\varphi_{z},\varepsilon_{x},\varepsilon_{y},\varepsilon_{z},\delta t_{u},\delta t_{ru}\right]}^{T},$$

where the 17 states are position errors, velocity errors, accelerometer drifts, attitude errors, gyroscope random drifts, satellite clock error $\delta t_{u}$ and clock drift $\delta t_{ru}$. Among them, the first 15 states are the parameters of INS, while the 16th and 17th dimensions are state errors of the GNSS receiver.

The measurements are the differences between the predicted (*I_INS_* and *Q_INS_*) and measured (*I_GNSS_* and *Q_GNSS_*) I/Q values. Typically, the observation matrix is defined as

(8) $$H_{k}={\left[\begin{array}{cccccccccc} h_{Ix1} & h_{Iy1} & h_{Iz1} & 0 & 0 & 0 & 0 & \cdots & 1 & 0\\ \vdots & \vdots & \vdots & \vdots & \vdots & \vdots & \vdots & \cdots & \vdots & \vdots \\ h_{Ixn} & h_{Iyn} & h_{Izn} & 0 & 0 & 0 & 0 & \cdots & 1 & 0\\ 0 & 0 & 0 & h_{Q\dot{x}1} & h_{Q\dot{y}1} & h_{Q\dot{z}1} & 0 & \cdots & 0 & 1\\ \vdots & \vdots & \vdots & \vdots & \vdots & \vdots & \vdots & \cdots & \vdots & \vdots \\ 0 & 0 & 0 & h_{Q\dot{x}n} & h_{Q\dot{y}n} & h_{Q\dot{z}n} & 0 & \cdots & 0 & 1 \end{array}\right]}_{2n\times 17},$$

where the elements in the matrix can be computed by

(9) $$h_{Ixn}=\frac{1}{2}\left(\frac{\partial E\left[I\right]}{\partial \theta_{e}}\frac{\partial \theta_{e}}{\partial x}+\frac{\partial E\left[I\right]}{\partial \omega_{e}}\frac{\partial \omega_{e}}{\partial x}\right),$$

(10) $$h_{Q\dot{x}n}=\frac{1}{2}\left(\frac{\partial E\left[Q\right]}{\partial \theta_{e}}\frac{\partial \theta_{e}}{\partial \dot{x}}+\frac{\partial E\left[Q\right]}{\partial \omega_{e}}\frac{\partial \omega_{e}}{\partial \dot{x}}\right).$$

It is important to note that Formulas (9) and (10) are described in *x* direction as an example, and the expressions in the other two directions share the similar form. Further calculations of the differentials defined in Formulas (9) and (10) can be carried out as follows

(11) $$\frac{\partial E\left[I\right]}{\partial \theta_{e}}=\frac{A}{2\omega_{e}}\left[\cos\left(\omega_{e}\left(k+1\right)T+\theta_{e}\right)-\cos\left(\omega_{e}kT+\theta_{e}\right)\right];$$

(12) $$\begin{array}{l} \frac{\partial E\left[I\right]}{\partial \omega_{e}}=-\frac{A}{2\omega_{e}^{2}}\left[\sin\left(\omega_{e}\left(k+1\right)T+\theta_{e}\right)-\sin\left(\omega_{e}kT+\theta_{e}\right)\right]\\ \;+\frac{A}{2\omega_{e}}\left[\left(k+1\right)T\cdot \cos\left(\omega_{e}\left(k+1\right)T+\theta_{e}\right)-kT\cdot \cos\left(\omega_{e}kT+\theta_{e}\right)\right] \end{array};$$

(13) $$\frac{\partial E\left[Q\right]}{\partial \theta_{e}}=-\frac{A}{2\omega_{e}}\left[-\sin\left(\omega_{e}\left(k+1\right)T+\theta_{e}\right)+\sin\left(\omega_{e}kT+\theta_{e}\right)\right];$$

(14) $$\begin{array}{l} \frac{\partial E\left[Q\right]}{\partial \omega_{e}}=\frac{A}{2\omega_{e}^{2}}\left[\cos\left(\omega_{e}\left(k+1\right)T+\theta_{e}\right)-\cos\left(\omega_{e}kT+\theta_{e}\right)\right]\\ \;-\frac{A}{2\omega_{e}}\left[-\left(k+1\right)T\cdot \sin\left(\omega_{e}\left(k+1\right)T+\theta_{e}\right)+kT\cdot \sin\left(\omega_{e}kT+\theta_{e}\right)\right] \end{array};$$

(15) $$\frac{\partial \theta_{e}}{\partial x}=-\frac{\omega x_{e}}{cR_{e}},\frac{\partial \theta_{e}}{\partial y}=-\frac{\omega y_{e}}{cR_{e}},\frac{\partial \theta_{e}}{\partial z}=-\frac{\omega z_{e}}{cR_{e}};$$

(16) $$\frac{\partial \omega_{e}}{\partial x}=0,\frac{\partial \omega_{e}}{\partial y}=0,\frac{\partial \omega_{e}}{\partial z}=0;$$

(17) $$\frac{\partial \theta_{e}}{\partial \dot{x}}=\frac{\omega {\dot{x}}_{e}T}{cv_{e}},\frac{\partial \theta_{e}}{\partial \dot{y}}=\frac{\omega {\dot{y}}_{e}T}{cv_{e}},\frac{\partial \theta_{e}}{\partial \dot{z}}=\frac{\omega {\dot{z}}_{e}T}{cv_{e}};$$

(18) $$\frac{\partial \omega_{e}}{\partial \dot{x}}=\frac{\omega {\dot{x}}_{e}}{cv_{e}},\frac{\partial \omega_{e}}{\partial \dot{y}}=\frac{\omega {\dot{y}}_{e}}{cv_{e}},\frac{\partial \omega_{e}}{\partial \dot{z}}=\frac{\omega {\dot{z}}_{e}}{cv_{e}}.$$

In this section, the conventional model of centralized ultra-tight integration is introduced, and the widely used calculation process of Jacobian matrix is described by Formulas (11)–(18). In the next section, the problem of this conventional model will be discussed and a novel linearization method to compute Formulas (9) and (10) will be proposed.

## 3. Linearization Method for Observation Equation

In typical centralized ultra-tight integration, original multi-step data conversion needs to be performed by just one Kalman observation equation, which leads to extreme complexity and strong nonlinearity of the model. Therefore, the filtering results depend significantly on the accuracy of measurement model and the processing of non-linearity.

Currently, most researches are based on the observation equation expressed by Formulas (8)–(18) [19,20,21], but in actual integration, the strong nonlinearity implicated in such equation can easily cause filtering divergence. When using the extended Kalman filter, Jwo et al. [20,21] and Zhou et al. [27,28] regard Equations (9)–(18) as the calculation process of Jacobian matrix. It can be easily found that even though Formulas (9) and (10) relate I/Q signals with position and velocity errors by differential, it does not mean that the obtained expression is linear. In fact, not only are sines and cosines included in Formulas (11)–(18), but also the strong linearity caused by the fact that $\omega_{e}$ and $\theta_{e}$ can be expressed as the functions of $R_{e}$ and $v_{e}$, just as Equations (3) and (4).

This problem is noted by Chen et al. [26,30], but the linearization approach provided will make Formula (1) constant and then Equations (11) and (12) constant zero, which means that the I signal becomes unmeasurable. Through their work, it is also proved that the measurement model based on Equations (9)–(18) does not satisfy the stability condition, which is concluded as the essential reason of filter divergence. Therefore, according to their research, the north velocity and height are added as observation variables to ensure the integrity of measurement.

Herein, a novel linearization method is proposed to recalculate the Jacobian matrix of the measurement equation under the condition that I/Q signals are still measurable.

In essence, the errors of position and velocity are calculated from the amplitude difference between estimated and measured I/Q signals. After receiver loops enter the state of stable tracking, in other words the signal is locked precisely, I signal reaches the maximum amplitude and presents a binary distribution in a positive and negative amplitude value, while the amplitude of Q reaches its minimum, approximately the noise with random distribution. So, there comes a conclusion that compared with the amplitude of estimated I/Q signals (*I_INS_* and *Q_INS_*) and measured ones (*I_GNSS_* and *Q_GNSS_*), their absolute values are more meaningful. For the reason that a slight Doppler or carrier phase deviation may bring about a significant change on amplitude’s direction, the following forms of measurements are adopted here so that the signal burr and filtering instability caused by direct difference can be avoided.

(19) $$Z_{k}={\left[\left|I_{1}^{INS}\right|-\left|I_{1}^{GNSS}\right|,\cdots ,\left|I_{n}^{INS}\right|-\left|I_{n}^{GNSS}\right|,\left|Q_{1}^{INS}\right|-\left|Q_{1}^{GNSS}\right|,\cdots ,\left|Q_{n}^{INS}\right|-\left|Q_{n}^{GNSS}\right|\right]}^{T}.$$

So, the observation equation can be written as

(20) $$Z_{k}=\left[\begin{array}{c} Z_{k}^{1}\\ \vdots \\ Z_{k}^{n}\\ Z_{k}^{n+1}\\ \vdots \\ Z_{k}^{2n} \end{array}\right]=\left[\begin{array}{cccccc} h_{Ix1}\left(x_{e}\right) & h_{Iy1}\left(y_{e}\right) & h_{Iz1}\left(z_{e}\right) & 0 & 0 & 0\\ \vdots & \vdots & \vdots & \vdots & \vdots & \vdots \\ h_{Ixn}\left(x_{e}\right) & h_{Iyn}\left(y_{e}\right) & h_{Izn}\left(z_{e}\right) & 0 & 0 & 0\\ 0 & 0 & 0 & h_{Q\dot{x}1}\left({\dot{x}}_{e}\right) & h_{Q\dot{y}1}\left({\dot{y}}_{e}\right) & h_{Q\dot{z}1}\left({\dot{z}}_{e}\right)\\ \vdots & \vdots & \vdots & \vdots & \vdots & \vdots \\ 0 & 0 & 0 & h_{Q\dot{x}n}\left({\dot{x}}_{e}\right) & h_{Q\dot{y}n}\left({\dot{y}}_{e}\right) & h_{Q\dot{z}n}\left({\dot{z}}_{e}\right) \end{array}\right]+\left[\begin{array}{c} \delta t_{u}\\ \vdots \\ \delta t_{u}\\ \delta t_{ru}\\ \vdots \\ \delta t_{ru} \end{array}\right]+V_{k}.$$

Taylor expansion is carried out for the sines and cosines in Equations (11)–(18), and the second order term is reserved. The calculation of Equation (12) is unnecessary due to Equations (8) and (16).

(21) $$\frac{\partial E\left[I\right]}{\partial \theta_{e}}=-\frac{A}{4}\left[\left(2k+1\right)\omega_{e}T^{2}+2T\theta_{e}\right];$$

(22) $$\frac{\partial E\left[Q\right]}{\partial \theta_{e}}=\frac{AT}{2};$$

(23) $$\frac{\partial E\left[Q\right]}{\partial \omega_{e}}=\frac{A}{4}\left(2k+1\right)T^{2}.$$

Introducing Equations (3), (4), (15)–(18) and (21)–(23), and then for those components with *R_e_* or *v_e_* in denominator, Taylor expansions of position error or velocity error are needed on the corresponding direction. The results can be given by

(24) $$\begin{array}{l} h_{Ix1}\left(x_{e}\right)=\frac{1}{2}\left(\frac{\partial E\left[I\right]}{\partial \theta_{e}}\frac{\partial \theta_{e}}{\partial x}+\frac{\partial E\left[I\right]}{\partial \omega_{e}}\frac{\partial \omega_{e}}{\partial x}\right)\\ \;=-\frac{A}{8}\left[\left(2k+1\right)\omega_{e}T^{2}\cdot \frac{-\omega }{c\sqrt{y_{e}^{2}+z_{e}^{2}}}+2T\frac{\omega^{2}}{c^{2}}-2T^{2}\cdot \frac{\omega^{2}v_{e}}{c^{2}\sqrt{y_{e}^{2}+z_{e}^{2}}}\right]\cdot x_{e} \end{array}$$

(25) $$\begin{array}{l} h_{Q\dot{x}1}\left({\dot{x}}_{e}\right)=\frac{1}{2}\left[\frac{\partial E\left[Q\right]}{\partial \theta_{e}}\frac{\partial \theta_{e}}{\partial {\dot{x}}_{e}}+\frac{\partial E\left[Q\right]}{\partial \omega_{e}}\frac{\partial \omega_{e}}{\partial {\dot{x}}_{e}}\right]\\ \;=\left[\frac{A\omega T^{2}}{4c\sqrt{{\dot{y}}_{e}^{2}+{\dot{z}}_{e}^{2}}}+\frac{A}{8}\left(2k+1\right)T^{2}\cdot \frac{\omega }{c\sqrt{{\dot{y}}_{e}^{2}+{\dot{z}}_{e}^{2}}}\right]\cdot {\dot{x}}_{e} \end{array}$$

The linearization result of the observation equation in *x* direction is given by Equations (24) and (25) as an example, and the forms are similar for other directions. As a consequence, the observation equation is expressed as a linear equation of position errors and velocity errors. Then, a standard Kalman filter can be used directly. What is more important is that compared with general Jacobian calculation retaining the first-order, a specific component in Equation (24) retains the accuracy of second-order, which plays an important role in improving positioning precision.

## 4. Integration Model Based on Code Approximation

The deviation of the model on ionosphere delay, troposphere delay and clock bias has been considered in Zhou’s study [31]. He argues that the system functions reflect the relationships between navigation errors and expectations of I/Q, rather than I/Q themselves, but the problem of code phase error is still not considered.

The deviation of code loop is the main source of position errors and the foundation of precise measurements. However, since the model based on scalar code loops is intended to assist the carrier loop, the error of code tracking is not considered in view of the dynamics on the code signal being less [19]. Thus, not only is the code error not considered in I/Q prediction, but position error is also obtained from carrier initial phase. Hence, there is a big deviation between the position given by the system and the real value, which is not enough to accurately correct INS or to construct the feedback for vector tracking loops. So, a scalar code loop is still selected reluctantly to track the code phase, like Figure 1.

When I/Q signals are ideal, that is, the amplitude of I reaches the maximum while the Q reaches its minimum, the loops are stable and maintain tracking. In this condition, the error of code loop is small, and the positioning error caused by ignoring code error is not obvious. This also occurs in the scenario where the simulated I/Q signals are used.

Though real-sampled signals are used in Jwo’s semi-physical simulation, the I/Q signals are indirectly converted from real-sampled pseudo-ranges and pseudo-range rates [20,21]. Suppose the mathematical model between the error of pseudo-range δρ and code phase τ can be given by

(26) δρ=Kτ

where *K* converts from chips to meters. Pseudo-ranges and positions can be related by [16]

(27) $$\delta \rho =-u^{T}\left(R_{u}-{\hat{R}}_{u}\right),$$

where $u^{T}$ denotes the unit line-of-sight vector from receiver to satellite.

Combining Equation (27) with (4), the conversion from the errors of pseudo-ranges and pseudo-range rates to I/Q information can be carried out successfully. However, because the pseudo-range error itself contains the deviation of code loop, the code bias is reflected in carrier initial phase through the conversion process above. As a result, the precise value of position can be obtained from carrier initial phase, even though the code error has never been taken into account in the model. In other words, the problem caused by neglecting code error is covered, but this is not the case when real-sampled I/Q signals are used for experiments. The ignored code bias will accumulate in position estimation, which is especially obvious when code error is large or code loop does not maintain tracking and oscillates seriously.

**Figure 2:**
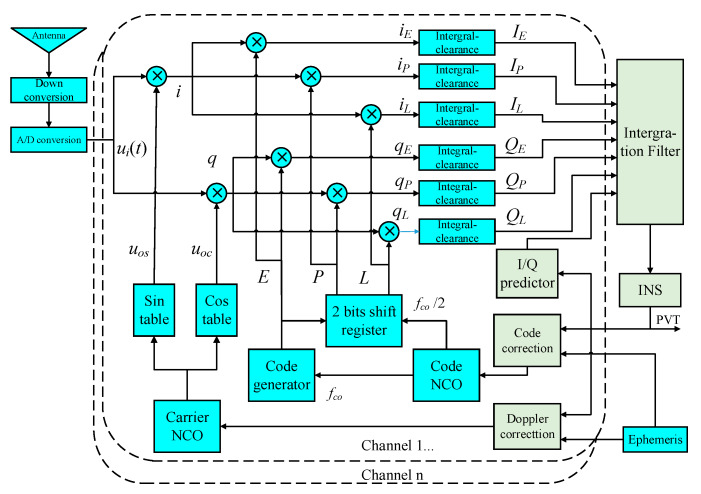
Ultra-tight integration considering code errors.

**Figure 3:**
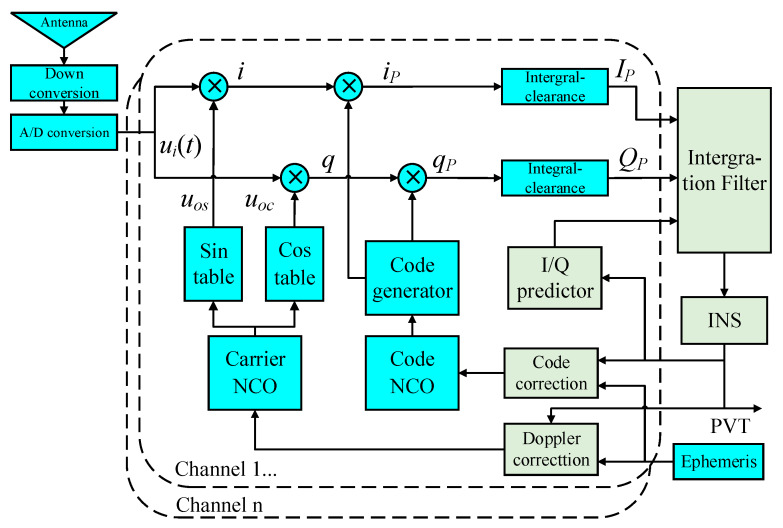
Ultra-tight integration considering code errors without new measurements.

As seen in the diagram in Figure 2, if the bias of code loop is considered to improve the model, not only the I/Q signals from prompt channel but also those from early and late branches should be involved for phase detecting. In this way, the number of observations for each channel will increase from two to six and the increase of filter dimension caused by new measurements will bring greater pressure to calculation. Therefore, a new approximate method to reduce the influence of code error, without introducing early and late measurements, is proposed herein. In this method, code delay and self-correlation function are approximated using position errors and are coupled to the I/Q predictions. As a result, conventional scalar code-tracking loop and its discriminator using *I_E_*/*Q_E_* and *I_L_*/*Q_L_* can be canceled. The architecture can be seen in Figure 3, and through this new model, it is capable of performing vector code-tracking due to the essential improvement on positioning accuracy.

Considering code phase error, the I/Q can be rewritten as follows

(28) $$I_{P}=\int_{kT}^{(k+1)T}\cos(\hat{\omega }t+\hat{\theta })\left[A\cos(\omega^{\prime }t+\theta^{\prime })+\eta \right]R\left(\tau \right)dt$$

(29) $$Q_{P}=\int_{kT}^{(k+1)T}\sin(\hat{\omega }t+\hat{\theta })\left[A\cos(\omega^{\prime }t+\theta^{\prime })+\eta \right]R\left(\tau \right)dt$$

where R(τ) represents the self-correlation function of pseudo-code. When the code phase error |τ| is 0 chip, the value of self-correlation function is 1, while when |τ| is greater than or equal to 1 chip, the function value is 0 and the rest is linear interpolation. This function can be approximately written as

(30) $$R\left(\tau \right)=1-\frac{\left|R_{e}\right|}{CP}$$

where *CP* denotes the length of a chip in pseudo-code. Accordingly, Formulas (1) and (2) can be given by

(31) $$E\left[I_{P}\right]=\frac{A}{2\omega_{e}}\left[\sin\left(\omega_{e}\left(k+1\right)T+\theta_{e}\right)-\sin\left(\omega_{e}kT+\theta_{e}\right)\right]\cdot \left(1-\frac{\left|R_{e}\right|}{CP}\right);$$

(32) $$E\left[Q_{P}\right]=-\frac{A}{2\omega_{e}}\left[\cos\left(\omega_{e}\left(k+1\right)T+\theta_{e}\right)-\cos\left(\omega_{e}kT+\theta_{e}\right)\right]\cdot \left(1-\frac{\left|R_{e}\right|}{CP}\right)$$

To avoid the differentiating of absolute value and introducing new nonlinear factors, the linearization result of the measurement equation can be approximately expressed as follows

(33) $$h_{Ix1}\left(x_{e}\right)=-\frac{A}{8}\left[\left(2k+1\right)\omega_{e}T^{2}\cdot \frac{-\omega }{c\sqrt{y_{e}^{2}+z_{e}^{2}}}+2T\frac{\omega^{2}}{c^{2}}-2T^{2}\cdot \frac{\omega^{2}v_{e}}{c^{2}\sqrt{y_{e}^{2}+z_{e}^{2}}}\right]\cdot \left(1-\frac{\sqrt{y_{e}^{2}+z_{e}^{2}}}{CP}\right)\cdot x_{e};$$

(34) $$h_{Q\dot{x}1}\left({\dot{x}}_{e}\right)=\left[\frac{A\omega T^{2}}{4c\sqrt{{\dot{y}}_{e}^{2}+{\dot{z}}_{e}^{2}}}+\frac{A}{8}\left(2k+1\right)T^{2}\cdot \frac{\omega }{c\sqrt{{\dot{y}}_{e}^{2}+{\dot{z}}_{e}^{2}}}\right]\cdot \left(1-\frac{\sqrt{y_{e}^{2}+z_{e}^{2}}}{CP}\right)\cdot {\dot{x}}_{e}.$$

The linearization result of the measurement equation in *x* direction is given by Equations (33) and (34) as an example, and other directions share the similar form. Consequently, the code tracking deviation is contained in not only the predicted I/Q signals generated by Equations (31) and (32), but also the position error obtained from measurements according to Equations (33) and (34). The covariance of measurement noise of the improved ultra-tight model is initialized by

(35) $$R=diag\left\{\begin{array}{cccccc} \sigma^{2}\left(I_{1}^{GNSS}\right) & \cdots & \sigma^{2}\left(I_{n}^{GNSS}\right) & \sigma^{2}\left(Q_{1}^{GNSS}\right) & \cdots & \sigma^{2}\left(Q_{n}^{GNSS}\right) \end{array}\right\},$$

where σ denotes the standard deviation.

## 5. State Equations and Stability of the Filter

### 5.1. State Equations of the Extended Kalman Filter

For GNSS/INS integrated systems, their process model can be described by the following error equations. Suppose L, λ, and h are latitude, longitude and height, respectively; the error equations of position can be given by

(36) $$\delta \dot{L}=\frac{\delta \dot{y}}{r_{n}+h}-\delta h\frac{\dot{y}}{{\left(r_{n}+h\right)}^{2}};$$

(37) $$\delta \dot{\lambda }=\frac{\delta \dot{x}}{r_{e}+h}\sec L+\delta L\frac{\dot{x}}{r_{e}+h}\tan L\sec L-\delta h\frac{\dot{x}\sec L}{{\left(r_{e}+h\right)}^{2}};$$

(38) $$\delta \dot{h}=\delta \dot{z},$$

where $r_{e}$ and $r_{n}$ are the radii of prime vertical and meridian circles, respectively. The error equations of velocity can be described by

(39) $$\begin{array}{l} \delta \ddot{x}=\varphi_{z}f_{y}-\varphi_{y}f_{z}+\delta \dot{x}\frac{\dot{y}\tan L-\dot{z}}{r_{e}+h}+\delta \dot{y}\left(2\omega_{ie}\sin L+\frac{\dot{x}\tan L}{r_{e}+h}\right)-\delta \dot{z}\left(2\omega_{ie}\cos L+\frac{\dot{x}}{r_{e}+h}\right)\\ \;+\delta L\left[2\omega_{ie}\left(\dot{z}\sin L+\dot{y}\cos L\right)+\frac{\dot{x}\dot{y}}{r_{e}+h}\sec^{2}L\right]+\delta h\frac{\dot{x}\dot{z}-\dot{x}\dot{y}\tan L}{{\left(r_{e}+h\right)}^{2}}+\nabla_{x} \end{array};$$

(40) $$\begin{array}{l} \delta \ddot{y}=-\varphi_{z}f_{x}+\varphi_{x}f_{z}-\delta \dot{x}\times 2\left(\omega_{ie}\sin L+\frac{\dot{x}\tan L}{r_{e}+h}\right)-\delta \dot{y}\frac{\dot{z}}{r_{n}+h}-\delta \dot{z}\frac{\dot{y}}{r_{n}+h}\\ \;-\delta L\left[2\dot{x}\omega_{ie}\cos L+\frac{{\dot{x}}^{2}}{r_{n}+h}\sec^{2}L\right]+\delta h\left[\frac{\dot{x}\dot{z}}{{\left(r_{n}+h\right)}^{2}}+\frac{{\dot{x}}^{2}\tan L}{{\left(r_{e}+h\right)}^{2}}\right]+\nabla_{y} \end{array};$$

(41) $$\begin{array}{l} \delta \ddot{z}=\varphi_{y}f_{x}-\varphi_{x}f_{y}+\delta \dot{x}\times 2\left(\omega_{ie}\cos L+\frac{\dot{x}}{r_{e}+h}\right)+\delta \dot{y}\frac{2\dot{y}}{r_{n}+h}\\ \;-2\delta L\cdot \dot{x}\omega_{ie}\sin L-\delta h\left[\frac{{\dot{y}}^{2}}{{\left(r_{n}+h\right)}^{2}}+\frac{{\dot{x}}^{2}}{{\left(r_{e}+h\right)}^{2}}\right]+\nabla_{z} \end{array},$$

where $\omega_{ie}$ is the rotation rate of the earth-centered earth-fixed frame (*e* frame) relative to the earth-centered inertial frame (*i* frame) and f is specific force. The error equations of attitude can be expressed as

(42) $${\dot{\varphi }}_{x}=\varphi_{y}\left(\omega_{ie}\sin L+\frac{\dot{x}}{r_{e}+h}\tan L\right)-\varphi_{z}\left(\omega_{ie}\cos L+\frac{\dot{x}}{r_{e}+h}\right)-\frac{\delta \dot{y}}{r_{n}+h}+\delta h\frac{{\dot{y}}^{2}}{{\left(r_{n}+h\right)}^{2}}-\varepsilon_{x};$$

(43) $${\dot{\varphi }}_{y}=-\varphi_{x}\left(\omega_{ie}\sin L+\frac{\dot{x}}{r_{e}+h}\tan L\right)-\varphi_{z}\frac{\delta \dot{y}}{r_{n}+h}-\delta L\cdot \omega_{ie}\sin L+\frac{\delta \dot{x}}{r_{e}+h}-\delta h\frac{\dot{x}}{{\left(r_{e}+h\right)}^{2}}-\varepsilon_{y};$$

(44) $${\dot{\varphi }}_{z}=\varphi_{x}\left(\omega_{ie}\cos L+\frac{\dot{x}}{r_{e}+h}\right)+\varphi_{y}\frac{\dot{y}}{r_{n}+h}+\delta L\left(\omega_{ie}\cos L+\frac{\delta \dot{x}}{r_{e}+h}\sec^{2}L\right)+\frac{\delta \dot{x}}{r_{e}+h}\tan L-\varepsilon_{z}.$$

Accelerometer drifts and gyro random drifts can be modeled as follows

(45) $$\varepsilon =\varepsilon_{b}+w_{g};$$

(46) $$\nabla =\nabla_{b}+w_{a},$$

where $\varepsilon ={\left[\varepsilon_{x},\varepsilon_{y},\varepsilon_{z}\right]}^{T}$,$\nabla =\left|\nabla_{x},\nabla_{y},\nabla_{z}\right|$.$\varepsilon_{b}$ and $\nabla_{b}$ are random constants, and $w_{g}$, $w_{a}$ the white noises. For tightly integrated systems, the clock error $\delta t_{u}$ and clock drift $\delta t_{ru}$, which can be neglected in loose and the proposed ultra-tight integration, can be modeled as follows

(47) $$\delta {\dot{t}}_{u}=\delta t_{ru}+w_{tu};$$

(48) $$\delta {\dot{t}}_{ru}=-\frac{1}{T_{f}}\delta t_{ru}+w_{tru},$$

where $T_{f}$ is set according to filtering period and $w_{tu}$, $w_{tru}$ are white noises.

### 5.2. Stability Analysis of Filtering

Next, the stability of extended Kalman filter newly designed for the ultra-tightly integrated system needs to be discussed. Suppose $T_{o}$ is a full rank transformation matrix, and $X_{k}^{o}$ is the state divided into observable and unobservable parts, where $X_{k}=T_{o}X_{k}^{o}$. Then the state equation and observation equation can be written as

(49) $$T_{o}X_{k}^{o}=\Phi T_{o}X_{k-1}^{o}+\Gamma W_{k-1};$$

(50) $$Z_{k}=HT_{o}X_{k}^{1}+V_{k},$$

namely can be given by

(51) $$X_{k}^{o}=\Phi^{o}X_{k-1}^{o}+\Gamma^{o}W_{k-1};$$

(52) $$Z_{k}=H^{o}X_{k}^{o}+V_{k},$$

where

(53) $$\Phi^{o}=T_{o}^{-1}\Phi T_{o}=\left[\begin{array}{cc} \Phi_{11}^{o} & 0\\ \Phi_{21}^{o} & \Phi_{22}^{o} \end{array}\right]\begin{array}{c} \}n_{o}\\ \}n-n_{o} \end{array};$$

(54) $$\Gamma^{o}=T_{o}^{-1}\Gamma =\left[\begin{array}{c} \Gamma_{1}^{o}\\ \Gamma_{2}^{o} \end{array}\right]\begin{array}{c} \}n_{o}\\ \}n-n_{o} \end{array};$$

(55) $$H^{o}=HT_{o}=[\begin{array}{cc} \underset{n_{o}}{\underset{︸}{H_{1}^{o}}} & \underset{n-n_{o}}{\underset{︸}{0}} \end{array}].$$

Thus, equation (51) and (52) can be expressed as

(56) $$\left[\begin{array}{c} X_{k1}^{o}\\ X_{k2}^{o} \end{array}\right]=\left[\begin{array}{cc} \Phi_{11}^{o} & 0\\ \Phi_{21}^{o} & \Phi_{22}^{o} \end{array}\right]\left[\begin{array}{c} X_{\left(k-1\right)1}^{o}\\ X_{\left(k-1\right)2}^{o} \end{array}\right]+\left[\begin{array}{c} \Gamma_{1}^{o}\\ \Gamma_{2}^{o} \end{array}\right]W_{k-1};$$

(57) $$Z_{k}=\left[\begin{array}{cc} H_{1}^{o} & 0 \end{array}\right]\left[\begin{array}{c} X_{k1}^{o}\\ X_{k2}^{o} \end{array}\right]+V_{k}.$$

Then, the system can be divided into two independent subsystems given by

Subsystem1:$\{\begin{array}{c} X_{k1}^{o}=\Phi_{11}^{o}X_{\left(k-1\right)1}^{o}+\Gamma_{1}^{o}W_{k-1}\\ Z_{k}=H_{1}^{o}X_{k1}^{o}+V_{k} \end{array}$;

Subsystem2:$X_{k2}^{o}=\Phi_{21}^{o}X_{\left(k-1\right)1}^{o}+\Phi_{22}^{o}X_{\left(k-1\right)2}^{o}+\Gamma_{2}^{o}W_{k-1}$.

Actually,$X_{k}^{o}$ is expressed as

(58) $${\left[\delta x-\delta \dot{x},\delta y-\delta \dot{y},\delta z-\delta \dot{z},\delta x+\delta \dot{x},\delta y+\delta \dot{y},\delta z+\delta \dot{z}\right]}^{T},$$

and we can find a full rank matrix

(59) $$T_{o}=\left[\begin{array}{cccccc} 1/2 & 0 & 0 & 1/2 & 0 & 0\\ 0 & 1/2 & 0 & 0 & 1/2 & 0\\ 0 & 0 & 1/2 & 0 & 0 & 1/2\\ -1/2 & 0 & 0 & 1/2 & 0 & 0\\ 0 & -1/2 & 0 & 0 & 1/2 & 0\\ 0 & 0 & -1/2 & 0 & 0 & 1/2 \end{array}\right].$$

As a consequence, the original system can be easily written as two completely observable subsystems. What need to be noted is that the matrix $T_{o}$ and $X_{k}^{o}$ are not unique. Besides, if the initial value of mean-square error is set as $P_{0}>0$, the criterion of stable filtering is satisfied, which proves the Kalman filter described by Equations (51) and (52) stable. Suppose

(60) $$P_{k}^{o}=E\left[\left(X_{k}^{o}-{\hat{X}}_{k}^{o}\right){\left(X_{k}^{o}-{\hat{X}}_{k}^{o}\right)}^{T}\right]=T_{o}^{-1}E\left[\left(X_{k}-{\hat{X}}_{k}\right){\left(X_{k}-{\hat{X}}_{k}\right)}^{T}\right]T_{o}^{-T}=T_{o}^{-1}P_{k}T_{o}^{-T};$$

(61) $$K_{k}^{o}=P_{k}^{o}H^{oT}R^{-1}=T_{o}^{-1}P_{k}T_{o}^{-T}{\left(HT_{o}\right)}^{T}R^{-1}=T_{o}^{-1}P_{k}H^{T}R^{-1}=T_{o}^{-1}K_{k},$$

where $P_{k}$ and $K_{k}$ are mean square error and gain matrix of original filter, then we have

(62) $$\left(I-K_{k}^{o}H^{o}\right)\Phi^{o}=T_{o}^{-1}\left(I-K_{k}H\right)\Phi T_{o}.$$

It can be concluded that the transition matrix of system Equations (51) and (52) is the similar transformation of the transition matrix in the original system. According to the quality of invariant eigenvalues in similar transformations, both the original Kalman filter and transformed system are asymptotic stable.

## 6. Experiment and Simulation

### 6.1. Design and Configuration of the Expriment

**Figure 4:**
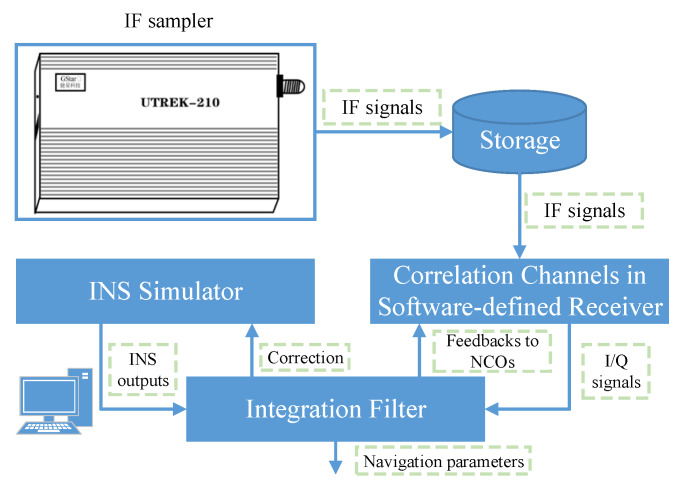
Schematic diagram for the experiment and simulation.

In order to verify the effectiveness of integration model based on code approximation and its linearization method for different loop status in a static environment, experiments of 700 s were carried out in the form of semi-physical simulation. The process of the experiment and simulation can be seen in Figure 4. The intermediate frequency (IF) data was obtained from an IF sampler, while the INS data was obtained from a simulator. The initial parameters of the simulation are all listed in Table 1.

The update frequencies of INS and I/Q measurements were 100 Hz and 1000 Hz, respectively, and the corresponding interval of coherent integration was 1 ms. Considering the balance between speed and precision, the extended Kalman filter was designed to run at 1 Hz.

### 6.2. Experiment and Analysis for Stable Tracking Loops

**Figure 5:**
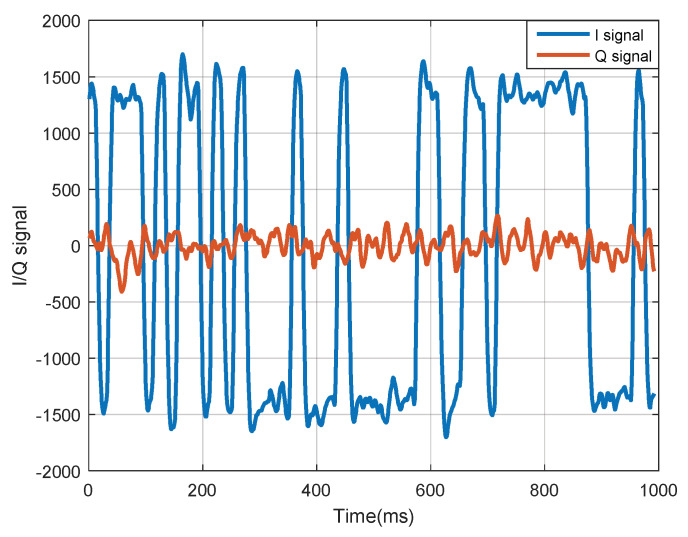
Coherent-accumulation measurement (I/Q) signals of GPS L1 from a stable tracking loop.

To verify the effectiveness of the lineaization method and the model based on code approximation, I/Q signals from stable tracking loops were used. The satellite signals were collected by an IF sampler, then mixed with carrier and code replicas generated by a receiver, where coherent integration interval was set to 1 ms so that the I/Q was obtained at 1000 Hz. Although only one satellite I/Q is necessary for this model, the signals from four satellites were still selected in simulation for the purpose of not losing generality. When the tracking loop was stable, the I/Q signals of GPS L1 are shown in Figure 5.

Figure 5 illustrates a maximum for the amplitude of I channel, which was approximately coincident with a binary distribution, while the amplitude of Q channel reached its minimum nearly as random noise. That demonstrates the conclusion that the loops in receiver have already entered the state of precise tracking.

**Figure 6:**
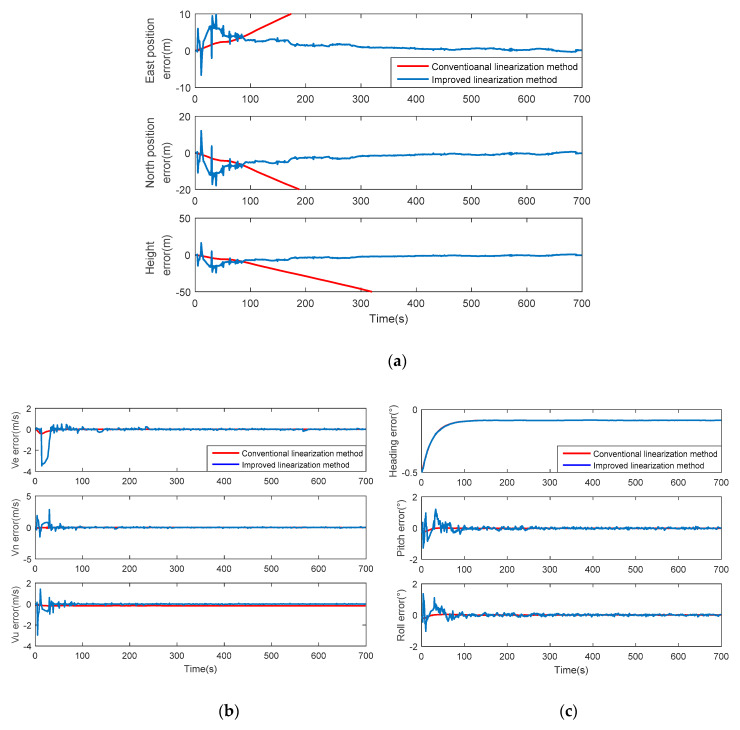
Comparison between conventional and improved linearization methods when tracking loop is stable. (**a**) Position errors; (**b**) velocity errors; (**c**) attitude errors.

The errors of various parameters are shown in Figure 6. When conventional observation equation and its linearization method proposed in [19,20,21,27,28,29] are used, the errors of velocity and attitude converge to zero well, and the curves are smooth. However, as indicated by the divergence of position errors, a strong nonlinearity is still implicated in the conventional model. As we can see from Formula (9)–(18), the errors of frequency and initial phase are functions of $x_{e}$ or ${\dot{x}}_{e}$ inherently. The Jacobian matrix obtained is extremely nonlinear, which leads to the large errors and filter divergence shown in Figure 6a. Compared with velocity, position seems to be more sensitive to non-linearity. However, by linearizing the measurement equation and calculating the Jacobian matrix following the novel approach provided in this paper, a completely linear expression can be achieved. As a result, a better convergence on position can be seen in Figure 6a, while the velocity and attitude error, which are still convergent, fluctuate slightly in an acceptable range. It is necessary to note that the display axis in Figure 6 is adjusted for a better view of the performance of improved method so that the diverged position errors in conventional method cannot be fully displayed.

The root-mean-square error (RMSE) and the standard deviation (STD) of those two linearization methods for observation equations are listed in Table 2. The RMSE indicates the overall size of errors, while the STD represents the dispersion degree. According to the table, a significant increase on position accuracy can be concluded in the improved linearization method, where the RMSE of east position reaches 0.8223 m. Contrarily, at the expense of improvement on position, there is a slight degeneration on velocity and attitude, with 0.0190 m/s RMSE for up-speed and 0.0455° RMSE for pitching. Moreover, the STD of velocity error and attitude error increased fractionally, indicating the increase of error fluctuation, but still remained within a small range.

### 6.3. Experiment and Analysis for Oscillatory Tracking Loops

**Figure 7:**
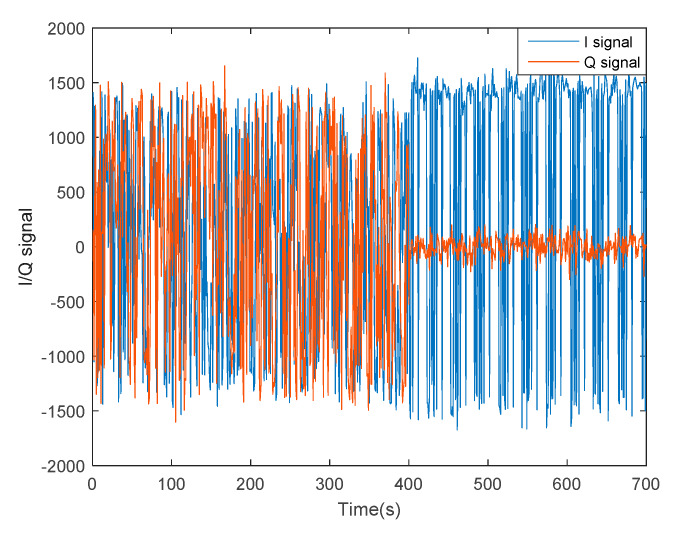
I/Q Signals of Beidou B1 from a stable oscillatory loop.

Compared with loose and tight integration, the advantages of an ultra-tight integration system are manifested as better loop tracking performance and system robustness. Therefore, a further test on system performance was carried out in the scenario where the tracking loop fails to track signals. As shown in Figure 7, I/Q signals of Beidou B1 fluctuate evidently between 0 and 400 s, illustrating that the tracking loop is in an oscillating state and the signal is not locked. After 400 s, Q is approximate to noise while I amplitude reaches the maximum, indicating a stable state of tracking loops. To avoid crowded curves and to describe the relationship of I and Q signals clearly, the signals shown in Figure 7 have been down-sampled.

**Figure 8:**
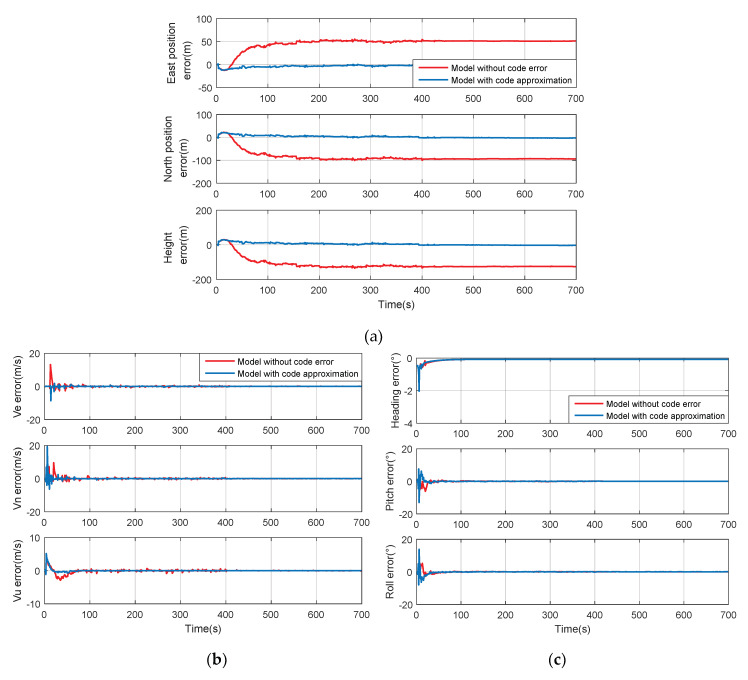
Comparison between the model without code error and with code approximation when tracking loop is oscillatory. (**a**) Position errors; (**b**) velocity errors; (**c**) attitude errors.

The system performance was tested under the condition described by Figure 7, and the conventional method ignoring code error and the improved method with code phase approximation were compared, as shown in Figure 8. Through these figures, a nearly equivalent performance on velocity and attitude can be seen, and all these errors converge to zero. However, there exists a large deviation between calculated position and real value in the traditional method. Though position errors eventually converge, it tends to accumulate with the oscillation of loops. As a result, the position error keeps approximately constant, and the feedbacks for INS and tracking loops all deviate.

In the conventional method, code delay is neglected. Assuming a scalar code-loop is used, only carrier parameters are considered in the integration filter. This decoupling may lead to a large position error and failing to correct INS accurately. This degradation is more obvious when the tracking loop is oscillating because the code delay is large and cannot be ignored any more. Before 400s, the INS is not corrected well and the error is growing, and this accumulated error is still unable to be corrected automatically after the loop becomes stable. This degradation can be significantly improved by the new model based on code approximation.

The numerical statistics are listed in Table 3. It illustrates an essential improvement on position precision in the new approach, where the RMSE of east position reaches 1.5223 m. In the meantime, the velocity precision is improved and the attitude is roughly equal. Thus, it is concluded that the performance of the improved method is much better than the traditional one, with higher accuracy and better robustness, especially under the circumstance of loop oscillation.

**Figure 9:**
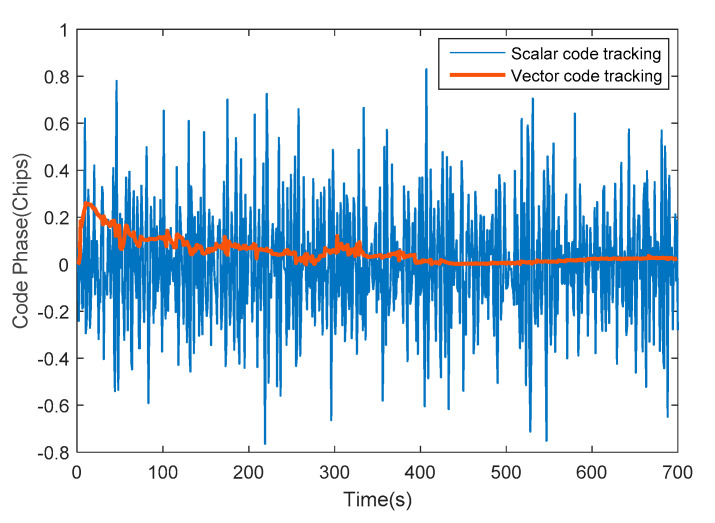
Comparison between scalar and the vector code tracking in the integrated system.

Except that it is difficult for the traditional method to build a vector code loop due to the position deviation, so the system still relies on a scalar one to a great extent. However, the vector tracking can be completely implemented using the improved method and the comparison is shown in Figure 9, which indicates the superiority and stability of vector feedback based on the new approach.

### 6.4. Comparison with Loosely and Tightly Integrated GNSS/INS Systems

To verify the superiority of the ultra-tight model based on code approximation and its novel linearization method, the comparison with loosely and tightly integrated GNSS/INS systems was conducted with the filters run at 1 Hz. In this experiment, the IF signal of Beidou B1 from a stable tracking loop was used, and the process model described in Section 6.1 was employed.

**Figure 10:**
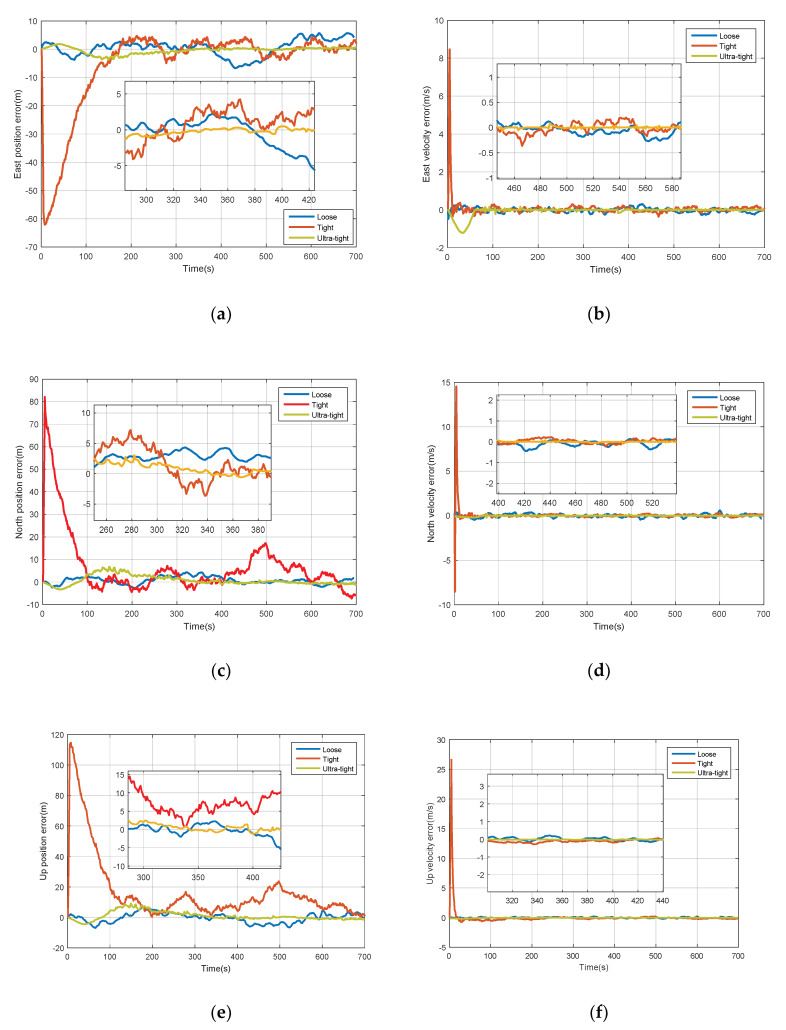
Comparison with loose and tight integration systems. (**a**) Position errors in east; (**b**) velocity errors in east; (**c**) position errors in north; (**d**) velocity errors in north; (**e**) position errors in up; (**f**) velocity errors in up.

When satellite signal is precisely tracked, the PVT (position, velocity and time) information can be calculated by the receiver, and the pseudo-ranges and pseudo-range rates can also be obtained. So, the loose, tight and ultra-tight integration are all able to be simulated, and the errors of various parameters are shown in Figure 10. Among them, the ultra-tightly integrated system demonstrates the best performance because the errors of position and velocity all converge to zero well with smoother curve and with less fluctuation. However, when the tracking loop is oscillating, navigation data cannot be demodulated so that neither the PVT nor the ephemeris can be obtained. As a result, the loose integration cannot be performed, and some tightly integrated architectures will be influenced because the estimated pseudo-range and pseudo-range rate is unavailable. In the scenario with higher vehicle dynamic, the superiority of the ultra-tightly coupled system in position and velocity errors would probably be more obvious. In a tightly integrated system, clock bias $\delta t_{u}$ and drift $\delta t_{ru}$ need to be taken into account when predicting pseudo-range and pseudo-range rate using the outputs of INS. Without any priori information, initial bias and drift were set far from the true values. There exists a process for $\delta t_{u}$ and $\delta t_{ru}$ to converge. Correspondingly, there is a rapidly rising error at the beginning of the tightly coupled system. Besides, the covariance of process noises of $\delta t_{u}$ and $\delta t_{ru}$ are also set to large values $Q_{t}=diag\left\{\begin{array}{cc} {\left(5\;m\right)}^{2} & {\left(0.1\;m/s\right)}^{2} \end{array}\right\}$, and this is why the precision of tight integration is not better than loose integration. Generally, $\delta t_{u}$ and $\delta t_{ru}$ need to be converted from time to distance using the speed of light.

## 7. Conclusions

There is a significant problem in an ultra-tight system with a centralized structure. The problem is the strong nonlinearity implicated in the measurement equation. Although the linearization of the model can be avoided in UKF and CKF frameworks, EKF is always selected as the comparison subject of those algorithms. However, the linearization methods or calculations of the Jacobian matrix in current studies are still not solved well, which means that the trouble in stability conditions and imperceptible nonlinear factors still need to be dealt with. Therefore, a novel approach to the linearize observation equation was put forward here. The method partly reserved two-order precision compared with commonly one-order in the Jacobian matrix. As a result, the filter converged well, and high positioning accuracy was achieved even though the performances of velocity and attitude are approximately the same as the original model, or slightly worse.

In the conventional method, when I/Q information is modeled with the errors of position and velocity, the code phase is ignored selectively and the position error given by the filter is estimated from the error of carrier initial phase. The large deviation caused by those problems may make the system fail to correct INS or construct the feedback of tracking loops. This phenomenon is more obvious when I/Q signals are not ideal, or the simulated ones are used. New measurements will be introduced inevitably if the error of code loop is taken into consideration, that is, the I/Q signals on early and late branches are necessary. However, that comes with great pressure that will be loaded on the integration filter. So, a new method with code phase approximation was proposed. The semi-physical simulation indicated a decline in the influence of code error, especially when the loops oscillated heavily. Consequently, the precision of positioning was greatly improved, while the accuracy of velocity and attitude always kept a high level.

## Figures and Tables

**Table 1:** Initial parameters in the simulation.

Items	Values
Initial position	(32.05856° N, 118.78864° E, 93 m)
Initial heading/pitch/roll	45°/0°/0°
Initial errors	0.5°/0.2°/0.2°(heading/pitch/roll)
Gyro constant drift	0.5°/h
Gyro random drift	0.1°/h
Accelerometer constant bias	0.3 mg
Accelerometer random bias	0.05 mg
Angular velocity range	0–400°/s
Acceleration range	0–4 g

**Table 2:** Numerical statistics and camparison of linearization methods (after 200 s).

Navigation Parameters	Root-Mean-Square Errors		Standard Deviation of Errors	
	Conventional Method	Improved Method	Conventional Method	Improved Method
East position (m)	32.1630	0.8223	10.8981	0.5029
North position (m)	58.5290	1.4964	19.8320	0.9152
Height (m)	78.8006	2.0147	26.7008	1.2322
East velocity (m/s)	0.0083	0.0442	0.0082	0.0442
North velocity(m/s)	0.0109	0.0352	0.0078	0.0349
Up velocity (m/s)	0.1700	0.0190	0.0051	0.0188
Heading (°)	0.0862	0.0862	0.0007	0.0007
Pitch (°)	0.0135	0.0455	0.0038	0.0437
Roll (°)	0.0114	0.0467	0.0054	0.0457

**Table 3:** Numerical statistics of traditional method and improved method (after 200 s).

Navigation Parameters	Root-Mean-Square Errors		Standard Deviation of Errors	
	Traditional Method	Improved Method	Traditional Method	Improved Method
East position (m)	51.1424	1.5223	0.9795	1.4656
North position (m)	93.0710	2.7702	1.7825	2.6671
Height (m)	125.3040	3.7297	2.3998	3.5908
East velocity (m/s)	0.1222	0.0694	0.1219	0.0694
North velocity (m/s)	0.1406	0.0645	0.1406	0.0645
Up velocity (m/s)	0.1276	0.0222	0.1277	0.0220
Heading (°)	0.0862	0.0862	0.0007	0.0007
Pitch (°)	0.0595	0.0516	0.0581	0.0508
Roll (°)	0.0448	0.0574	0.0441	0.0568
